# Genomic Analysis of Detoxification Supergene Families in the Mosquito *Anopheles sinensis*

**DOI:** 10.1371/journal.pone.0143387

**Published:** 2015-11-20

**Authors:** Dan Zhou, Xianmiao Liu, Yan Sun, Lei Ma, Bo Shen, Changliang Zhu

**Affiliations:** Department of Pathogen Biology, Nanjing Medical University, Nanjing, Jiangsu, 210029, P. R. China; Institute of Zoology, Chinese Academy of Sciences, CHINA

## Abstract

*Anopheles sinensis* is an important malaria vector in China and other Southeast Asian countries, and the emergence of insecticide resistance in this mosquito poses a serious threat to the efficacy of malaria control programs. The recently published *An*. *sinensis* genome and transcriptome provide an opportunity to understand the molecular mechanisms of insecticide resistance. Analysis of the *An*. *sinensis* genome revealed 174 detoxification genes, including 93 cytochrome P450s (P450s), 31 glutathione-S-transferases (GSTs), and 50 choline/carboxylesterases (CCEs). The gene number was similar to that in *An*. *gambiae*, but represented a decrease of 29% and 42% compared with *Aedes aegypti* and *Culex quinquefasciatus*, respectively. The considerable contraction in gene number in *Anopheles* mosquitoes mainly occurred in two detoxification supergene families, P450s and CCEs. The available *An*. *sinensis* transcriptome was also re-analyzed to further identify key resistance-associated detoxification genes. Among 174 detoxification genes, 124 (71%) were detected. Several candidate genes overexpressed in a deltamethrin-resistant strain (DR-strain) were identified as belonging to the CYP4 or CYP6 family of P450s and the Delta GST class. These generated data provide a basis for identifying the resistance-associated genes of *An*. *sinensis* at the molecular level.

## Introduction

Malaria is a major public health problem in tropical and subtropical regions [1, 2]. Indeed, according to the World Health Organization (WHO), an estimated 198 million people were at risk of malaria, which caused approximately 584 000 deaths worldwide in 2013 [3]. Malaria is transmitted via the bites of infected female *Anopheles* mosquitoes, which includes nearly 484 species, distributed in seven subgenera [4, 5]. *Anopheles sinensis* is one of the major malaria vector mosquitoes in East Asia, ranging from the Philippines to Japan. [6–8]. Increased attention has been paid to this species because of its wide geographic distribution, high density and modest susceptibility to malaria [9, 10].Recently, *vivax* malaria has re-emerged in the areas where *An*. *sinensis* was the main vector in central China and Korea [11, 12].

Mosquito vector control is one of the most effective measures to prevent and control malaria, which particularly relies on the use of insecticides [13]. Unfortunately, excessive and continuous use of insecticides has resulted in the development and rapid spread of resistance, which represents the major obstacle to malaria control and elimination [14]. *An*. *sinensis* has developed resistance to various classes of insecticides and this resistance increased strikingly during 1990s in malaria endemic areas in China [15, 16]. Resistance to insecticides in *An*. *sinensis* was also reported in Korea, which hampered effective malaria control [17, 18]. In the battle against malaria, insecticide resistance monitoring and management is a key element.

The evolution of insecticide resistance occurs through complicated mechanisms, typically requiring the interaction of multiple genes [19]. Knowledge of the molecular mechanism of insecticide resistance is a basic requirement for resistance management. The resistance mechanisms against insecticides are mainly classified into two major groups: increased metabolic detoxification and reduced target site sensitivity [20]. The detoxification enzymes typically linked to insecticide resistance mainly include three major supergene families: cytochrome P450 monooxygenases (P450s), glutathione S-transferases (GSTs) and carboxyl/cholinesterases (CCEs). P450s are involved in the resistance to almost all insecticides [21–23], GSTs are mainly involved in 1,1,1-trichloro-2,2-bis-(*p*-chlorophenyl)ethylene (DDT) and organophosphate (OP) resistance [24] and CCEs are mainly involved in OP and carbamate resistance [25]. These three detoxification supergene families are generally quite numerous in the process of environmental detoxification interactions and enzymatic defense against xenobiotics [26]. Although the important role of detoxification supergene families in the evolution of insecticide resistance is well studied, only a small subset of the detoxification genes has been previously described and analyzed in *An*. *sinensis*.

In this study, we utilized the published genomic sequence of *An*. *sinensis* [27] to fully characterize the detoxification supergene families. The comparative genomic analysis with other three major mosquito vectors of human diseases (*An*. *gambiae*, *Aedes aegypti* and *Culex quinquefasciatus*) and *Drosophila melanogaster* could form the basis for further studies on the origin and evolutionary patterns of these supergene families during their different and complex life cycles. The available transcriptomic data of *An*. *sinensis* was also re-analyzed to identify detoxification genes associated with insecticide resistance.

## Materials and Methods

### Gene identification, annotation and phylogenetic classification

To identify P450, GST, and CCE genes in *An*. *sinensis*, we scanned the *An*. *sinensis* whole genome sequencing database at the NCBI (BioProject Accession: PRJNA209295; http://www.ncbi.nlm.nih.gov/bioproject/PRJNA209295) using blastp with default parameters and using known detoxification genes from *D*. *melanogaster* [28–30], *An*. *gambiae* [31], *Ae*. *aegypti* [32] and *C*. *quinquefasciatus* [33] as a first step. Subsequently, the three groups of detoxification enzymes of *An*. *sinensis* were identified by the HMMER program (http://hmmer.janelia.org/) with the protein domains for P450s (PF00067), GSTs (PF00043 and PF02798) and CCEs (PF00135), as described in the Pfam database. The results of the two different approaches were then merged. The special characteristics of P450, GST, and CCE genes were finally applied to confirm their candidature. Insect P450 are generally about 500 amino acids long. The heme-binding domain and conserved region FXXGXXXCXG allows identification of putative P450 sequences [29]. The sequences of GST were verified for conserved protein length (about 200 amino acids), and the presence of a SNAIL/TRAIL motif [34]. The catalytic triad sequence (Ser-His-Glu) was used to identify CCEs [34]. Protein sequences of the detoxification genes were aligned using ClustalW and phylogenetic trees for all three detoxification supergene families were determined by the neighbor-joining method with distance bootstrap values (1000 replicates). P450s were classified and named according to the guidelines of the P450 nomenclature committee (http://drnelson.uthsc.edu/CytochromeP450.html). With respect to GST and CCE genes, however, rules for classification have not been clearly established. Sequence identity and phylogenetic relationship were the major criteria for the assignment of GSTs and CCEs to “class” and “clade”, respectively. The gene orthology predictions were generated by using Ensemble Gene Tree method. This method was based on the algorithm PHYML for multiple protein sequence alignments generated using MUSXLE for each gene family containing sequences among mosquito species and *D*. *melanogaster*. Gene trees were reconciled with the species trees using the RAL algorithm to call duplication events on internal nodes and to root the trees. According to the results of each gene tree, the relations of orthology were inferred [35].

### Transcriptomic analyses of resistance-associated detoxification genes

The available *An*. *sinensis* transcriptome was re-analyzed for insecticide resistance-associated detoxification genes [27]. In the previous study, the field population of *An*. *sinensis* was collected from Shifosi (N29.95, E115.62) town of Hubei Province in 2011. After 2 to 3 day post adult emergence, non-blood female adult mosquitoes were phenotyped for susceptibility to 0.05% deltamethrin, using the standard WHO tube susceptibility bioassay, and were subsequently grouped as deltamethrin-susceptible strain (DS-strain) and deltamethrin-resistant strain (DR-strain). The mosquitoes which knocked down after one-hour exposure were classified as DS-strain, and those survived after the 24-hour recovery period were classified as DR-strain. Two libraries (DS-strain and DR-strain) were constructed to provide transcriptomic data to assess the assembly quality of the *An*. *sinensis* genome (BioProject Accession: PRJNA293400; http://www.ncbi.nlm.nih.gov/Traces/wgs/?val=GDKS01). In the present study, unigenes extracted from the transcriptome were BLAST searched against the identified *An*. *sinensis* detoxification genes with default parameters. A cutoff e-value of 1e-5 was used. Genes were identified as differentially expressed if they exhibited two-folds or greater changes between the DS-strain and DR-strain (|log2Ratio| ≥ 1), statistical significance at *P* < 0.001 and had a false discovery rate (FDR) ≤ 0.001.

## Results and Discussion

### Detoxification supergene families

After merging the gene sets generated by Blastp and HMMER, 184 detoxification sequences were identified. A manual review identified the vast majority of these sequences as full-length genes, although 24 sequences were identified as partial detoxification genes with high similarities to P450s or CCEs. These partial sequences usually locate at the start of scaffold, next to the internal gap and probably a result of assembly errors. Two truncated GST sequences were identified as C-terminal and N-terminal fragments of the same protein, respectively. The same situation also existed in the other sixteen truncated P450 sequences. Thus, the number of detoxification genes in *An*. *sinensis* was ultimately determined as 174, including 93 P450, 31 GST and 50 CCE genes (Table 1).

**Table 1:** Number and class distribution of detoxification genes in *Anopheles sinensis*, *Anopheles gambiae*, *Aedes aegypti*, *Culex quinquefasciatusin* and *Drosophila melanogaster*.

	Classification	*An*. *sinensis*	*An*. *gambiae*	*Ae*. *aegypti*	*C*. *quinquefasciatus*	*D*. *melanogaster*
P450	CYP2 clan	8	10	11	14	6
	CYP3 clan	44	42	84	88	36
	CYP4 clan	34	45	59	83	32
	Mitochondrial clan	7	9	10	11	11
GST	Delta class	12	12	8	14	11
	Epsilon class	7	8	8	10	14
	Omega class	1	1	1	1	5
	Sigma class	1	1	1	1	1
	Theta class	2	2	4	6	4
	Zeta class	1	1	1	0	2
	Unclassified class	7	3	3	3	0
CCE	B clade	22	16	22	30	13
	D clade	0	0	0	1	3
	E clade	2	4	2	3	2
	F clade	7	6	7	13	3
	G clade	4	4	6	9	0
	H clade	4	10	7	6	5
	I clade	1	1	1	1	1
	J clade	2	2	2	2	1
	K clade	1	1	1	1	1
	L clade	5	5	5	3	4
	M clade	2	2	2	2	2
Total		174	185	245	302	157

Data of *A*. *gambiae*, *A*. *aegypti* and *D*. *melanogaster* were taken from Oakeshott et al. [26]

Data of *C*. *quinquefasciatus* was taken from Yan et al. [33]

Then, we matched these identified detoxification genes against *An*. *sinensis* transcriptomic data (adult females only). Among 174 detoxification genes, 29% of the genes were neither detected in the DS-strain nor in the DR-strain, which could be explained as male- or juvenile-specific genes or untranscribed pseudogenes. As shown in Table 1, 73 (78%) P450s, 25 (81%) GSTs and 26 (52%) CCEs were expressed in at least one library. This result appeared similar to another study showing that 77% of P450s, 83% of GSTs and 65% of CCEs in *C*. *quinquefasciatus* could be identified in any life stage (egg, larva, pupa or male/female adult) [33]. In these two mosquito species, both P450s and GSTs exhibited a relatively high detectable rate with the exception of CCEs. Possibly because there are high numbers of CCE pseudogenes distributed in the whole mosquito genome. These pseudogenes are present but unable to function. It’s also possible that some CCEs may be usually in silent and could not be transcribed. Only when in response to a particular stimulation, their transcription and translation could be activated.

### Cytochrome P450s

#### Genome level analysis of P450s

The phase I detoxification enzymes, P450s, are involved in the metabolism of a diverse array of endogenous and xenobiotic compounds [36]. P450s constitute one of the largest and oldest gene superfamilies in insects. The functional and evolutionary diversification of P450s has contributed to the success of insects to adapt to almost every ecological environment [37].

**Fig 1:**
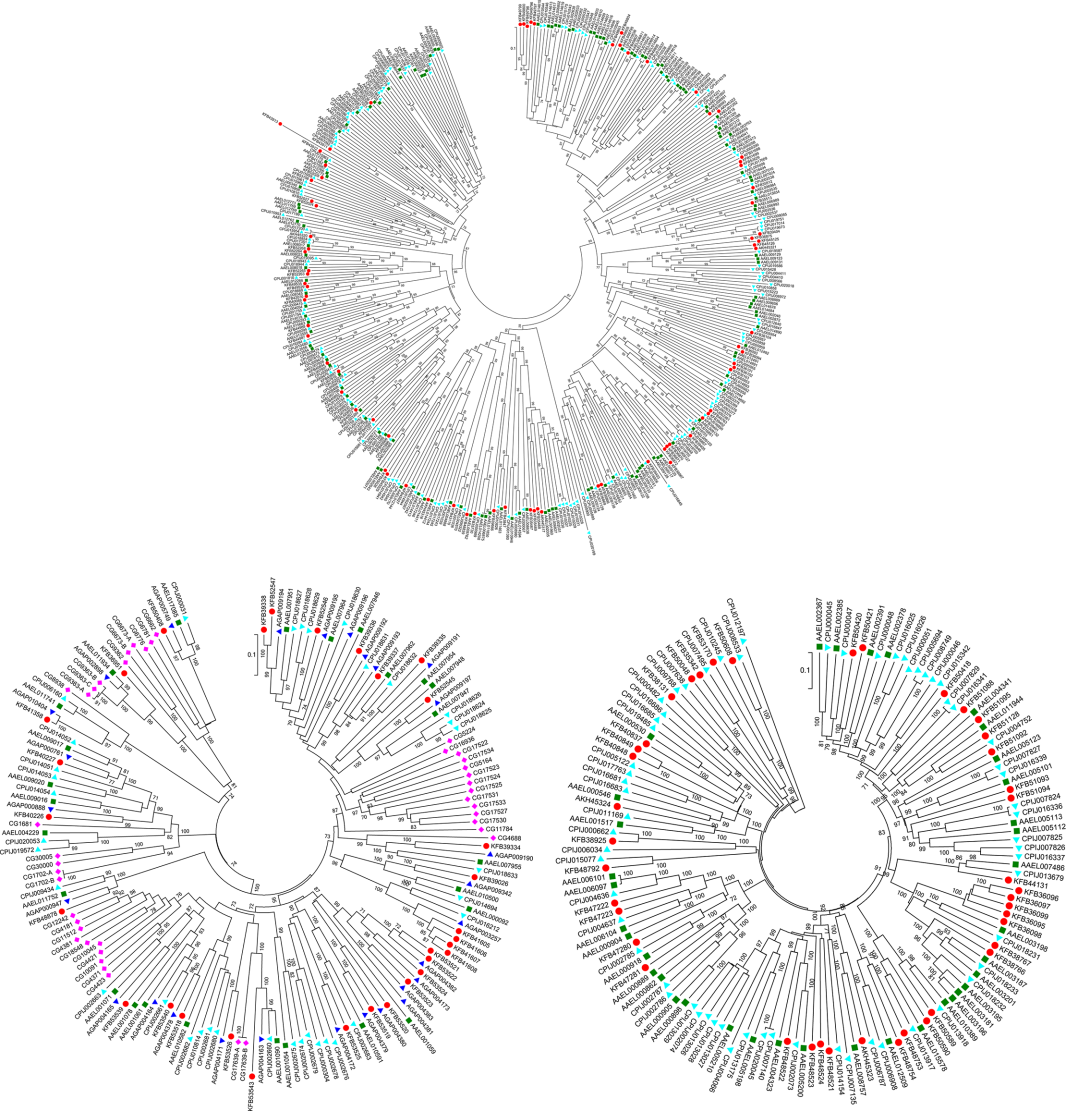
The phylogenetic analysis of cytochrome P450s, glutathione-S-transferases and choline/carboxylesterases. (A) Unrooted distance neighbor-joining tree showing the phylogeny of cytochrome P450s from the genomes of *Anopheles sinensis* (red circle), *Aedes aegypti* (green square) and *Culex pipiens quinquefasciatusin* (aqua triangle). (B) Unrooted distance neighbor-joining tree showing the phylogeny of glutathione-S-transferases from the genomes of *Anopheles sinensis* (red circle), *Anopheles gambiae* (blue triangle), *Aedes aegypti* (green square), *Culex pipiens quinquefasciatusin* (aqua triangle) and *Drosophila melanogaster* (pink rhombus). (C) Unrooted distance neighbor-joining tree showing the phylogeny of choline/carboxylesterases from the genome of *Anopheles sinensis* (red circle), *Aedes aegypti* (green square) and *Culex pipiens quinquefasciatusin* (aqua triangle). The percentage of bootstrap confidence values greater than 70% (1000 replicates) is indicated at the nodes.

All the identified P450s were classified and named according to the guidelines of the Cytochrome P450 nomenclature committee, using standard conventions for this gene superfamily (S1 Table). The phylogenetic trees of P450 sequences were constructed based on their consensus sequences (Fig 1A). The P450 clans are higher order groupings of P450 families. P450 genes within the same clan have likely diverged from a single gene ancestor [38]. The 93 P450s identified in *An*. *sinensis* were gathered into four distinct clans, CYP3, CYP4, CYP2 and mitochondrial CYP. There were 44 P450s in CYP3 (47%), 34 in CYP4 (37%), 8 in CYP2 (9%) and 7 in the Mitochondrial CYP clan (8%). As in other dipteran insects, the majority of the P450s were represented by the CYP3 and CYP4 clans, and each accounted for about 35–45% of the total P450 genes [26]. These two clans in Diptera appeared to have undergone significant species-specific radiations.

There were 44 CYP3 clan sequences in *An*. *sinensis*, which was similar to the numbers found in *An*. *gambiae*, but just half of that in *Ae*. *aegypti* and *C*. *quinquefasciatus*. *An*. *sinensis* has two CYP3 clan families: CYP6 and CYP9.

The CYP6 family is insect specific and evolutionary related to vertebrate CYP3 and CYP5 families [29, 39]. Thirty-one CYP6 genes could be further classified into 14 subfamilies. It should be noted that there was one novel subfamily, with a single sequence, CYP6HP1. The top BLAST hit for CYP6HP1 was *An*. *gambiae* CYP6R1v1. *An*. *sinensis* CYP6HP1 has only 51% identity with *An*. *gambiae *CYP6R1v1; therefore, it was difficult to predict whether the function was conserved between these two genes. Most of the subfamilies had one or two members. Expansion was observed in two subfamilies, CYP6M and CYP6Z, both containing five genes. Within the CYP6M subfamily, three out of five genes had orthologs in *An*. *gambiae*. However, no clear ortholog to the *An*. *sinensis* CYP6Z genes was identified in *An*. *gambiae*. The loss of CYP6R genes in *An*. *sinensis* was observed while this subfamily was conserved throughout the *Anopheles* mosquitoes [40].

Like CYP6, the CYP9 family contains only insect P450s. Thirteen CYP9 genes could be divided into four subfamilies: 9J, 9K, 9L and 9M. A majority of the CYP9 genes were in CYP9J subfamily, accounting for 60% of the CYP9 family. These CYP9J genes were physically clustered in the same scaffold (scf7180000696055). These increased amounts of tandem duplications leading to the expansion of CYP9J subfamily compared with *An*. *gambiae*. None of the CYP9J genes had orthologs in *An*. *gambiae*.

CYP4 was the second biggest clan in *An*. *sinensis*, comprising 34 members and could be arranged into two insect specific families (CYP4 and CYP325) and 16 subfamilies. However, the distribution of P450 members across the four clans in *An*. *gambiae* was inconsistent with three other mosquito species, as *An*. *gambiae* has slightly fewer members in the CYP3 clan and CYP4 is the biggest clan on its genome.

CYP4 family members account for fully 65% of the *An*. *sinensis* CYP4 clan. Nineteen out of twenty-two members in this family have orthologous genes in *An*. *gambiae*, which suggested a similar role for the CYP4 family in the two *Anopheles* mosquitoes. Among them, two *An*. *sinensis* genes (GenBank ID: KFB44985 and KFB44986) were co-orthologous to CYP4J9 in *An*. *gambiae*. The CYP4C is the largest subfamily, containing six members. Members of the insect CYP4G subfamily are notable for an unusually long insertion between helices F and G and a nontraditional N-terminal sequence. Compared with *An*. *gambiae*, a relatively large number of CYP4H losses in *An*. *sinensis* was observed (e.g. CYP4H15, 16, 18, 19, 24, 16 and 27), which resulted in the contraction of this subfamily.

The CYP325 family could be divided into two groups: one comprising subfamilies 325B, 325C and 325K, and another comprising subfamilies 325A, 325F, 325G, 325H and 325J. Compared with *An*. *gambiae*, CYP325D and CYP325E were lost in *An*. *sinensis*. The absence of CYP325D was also observed previously in *An*. *albimanus* and *An*. *merus* [40].

The CYP2 clan encompasses approximately 5.5–10% of the total P450s in most insects. In *An*. *sinensis*, there are eight CYP2 members (9%) arranged into five families, with 1–2 members in each family. The CYP2 members are fairly well conserved across the Diptera with limited examples of lineage specific duplications or losses. For example, CYP18A1, a conserved gene throughout the *Anopheles* mosquitoes (including *An*. *sinensis*), was not detected in any member of the *An*. *gambiae* complex [40].

To date, mitochondrial CYPs have only found in animals, and not in fungi or plants [41]. The microsomal CYP is a minor group among the total CYP family members of animals. The percentage of mitochondrial CYPs in the *Anopheles* mosquitoes (8%) was slightly more than the 6% in *Aedes* and *Culex* mosquitoes. The *An*. *sinensis* mitochondrial clan comprises CYP12, CYP302, CYP314 and CYP315 families. In seven mitochondrial CYPs, four genes belonged to CYP12F. The remaining mitochondrial CYPs (CYP302A1, 314A1 and 315A1), which are of unknown function, were originally thought to have 1:1:1 orthologies in the honeybee, mosquito and fruit fly [42]. However, these three mitochondrial CYPs in *An*. *sinensis* lacked clear orthologies to the above species. In addition, the CYP315 family in *C*. *quinquefasciatus* has not been identified [33].

#### Resistance associated P450s

According to the comparative transcriptomic results, of the five overexpressed P450s in the *An*. *sinensis* DR-strain, four P450s were represented by the CYP6 family and the remaining one was from CYP4 family (Table 2). These overexpressed CYP6 genes have been previously reported to be responsible for the resistance to insecticides in other mosquito species. For example, CYP6P2 was recently found to be overexpressed in bendiocarb resistant *An*. *gambiae* [22]. The over-transcription of CYP6AA1 and CYP6M3 in *An*. *gambiae* was associated with pyrethroids/DDT and dieldrin resistance [43]. CYP6M7 in *An*. *funestus* (the ortholog of CYP6M3) was located in the genomic region spanning the pyrethroid resistance *rp2* QTL and considered responsible for extending pyrethroid resistance [44]. Overexpression of these CYP6 genes linked repeatedly with insecticide resistance phenotype suggested a common feature in detoxification of insecticide in mosquito populations, which may provide potential candidates for P450-mediated insecticide resistance monitoring and management in *An*. *sinensis*. KFB40666, *An*. *gambiae* CYP4H14 ortholog, showed the largest increase in transcription in the *An*. *sinensis* DR-strain. Although other members in the subfamily CYP4H have been implicated in DDT resistance in *An*. *gambiae* [45] and pyrethroid resistance in *Ae*. *albopictus* [46], CYP4H14 has not been reported to be involved in insecticide-resistance. Its role in mosquito insecticide resistance required further investigation.

**Table 2:** Differential expression of *Anopheles. sinensis* detoxification genes between deltamethrin-susceptible and -resistant strains.

	Protein	NCBI_ID	Classification	log2(resistant/ susceptible)
P450	scf7180000695742.43	KFB40666	CYP4H14	9.18
	scf7180000695236.50	KFB36093	CYP6AA1	5.59
	scf7180000695502.5	KFB36870	CYP6M17	3.45
	scf7180000695935.4	KFB42894	CYP6M3	3.40
	scf7180000695236.60	KFB36103	CYP6P2	2.67
	scf7180000695685.3	KFB39402	CYP6Y2	-5.63
	scf7180000695502.6	KFB36871	CYP6M18	-4.32
	scf7180000695502.2	KFB36867	CYP6M1	-3.28
	scf7180000696055.157	KFB49805	CYP9L	-2.84
	scf7180000696055.156	KFB49804	CYP9L	-2.84
GST	scf7180000696131.148	KFB53540	Delta	6.19
	scf7180000696131.147	KFB53539	Delta	1.51
	scf7180000695709.152	KFB40227	Theta	-1.49
CCE	scf7180000695675.2	-	L	-4.39
	scf7180000696049.274	KFB48754	E	-1.66

We also identified five P450s expressed at lower levels in transcription in the *An*. *sinensis* DR-strain. It has been suggested that down-regulation of P450s may play roles in insecticide resistance because mosquitoes need to protect the cells from the deleterious effects of up-regulated P450s and thus balance the usage of energy, O_2_, or other components needed for the syntheses proteins [47–49]. Thus, we hypothesize that these poorly expressed P450s may be linked with adaptive or homeostatic response, which would be an advantage in the insecticide resistant mosquitoes. That may offer part of the explanation for why the expression profiles were different among the members in the gene-expanded cluster, CYP6Ms.

### Glutathione-S-transferases

#### Genome level analysis of GSTs

Thirty-one GST genes were identified in *An*. *sinensis*, which are approximately 10% and 19% gene-expanded compared with *An*. *gambiae* and *Ae*. *aegypti*, respectively, while similar gene numbers were found in *C*. *quinquefasciatus* (S2 Table). Their classification was performed based on sequence homology and phylogenetic relationships with the known GSTs (Fig 1B) [26]. The identified *An*. *sinensis* GSTs could be divided into seven classes: Delta, Epsilon, Omega, Sigma, Theta, Zeta and Unclassified. The Unclassified class is absent from *Drosophila* and the Zeta class is absent from *Culex*, while each of the GST classes was found in *Aedes* and the two *Anopheles* mosquitoes.

The GST supergene family belongs to the phase II detoxification system, which conjugates endogenous and xenobiotic toxins with electrophilic centers to glutathione [50]. In *An*. *sinensis*, over half of the GSTs belonged to the Delta (12, 39%) and Epsilon (7, 23%) classes, which were also the two largest classes of GSTs in other mosquitoes and in the fruit fly. However, the proportion of the Delta class in non-dipteran insects were relatively smaller, such as 8% in *Tribolium castaneum* (Coleoptera) [51] and 17% in *Bombyx mori* (Lepidoptera) [52]. In some hymenopteran insect orders, such as *Apis mellifera* and *Nasonia vitripennis*, no Epsilon class GSTs have been identified [26]. The expansion of the Delta and Epsilon classes in dipteran insects possibly occurred independently after the split between the dipteran and non-dipteran insects, presumably in response to diverse aspects of biology, and satisfying the specific needs of dipterans during adaptation to different environmental challenges. Within the *An*. *gambiae* Delta and Epsilon GST classes, there is evidence of recent internal duplications within gene clusters [53]. In the *An*. *sinensis* genome, 12 Delta GSTs are in the same scaffold (scf7180000696056) and seven Epsilon GSTs are arranged in two scaffolds (scf7180000696106 and scf7180000695681), suggesting the expansion of Delta and Epsilon classes in *An*. *sinensis* may be partly the result of local gene duplications.

**Fig 2:**
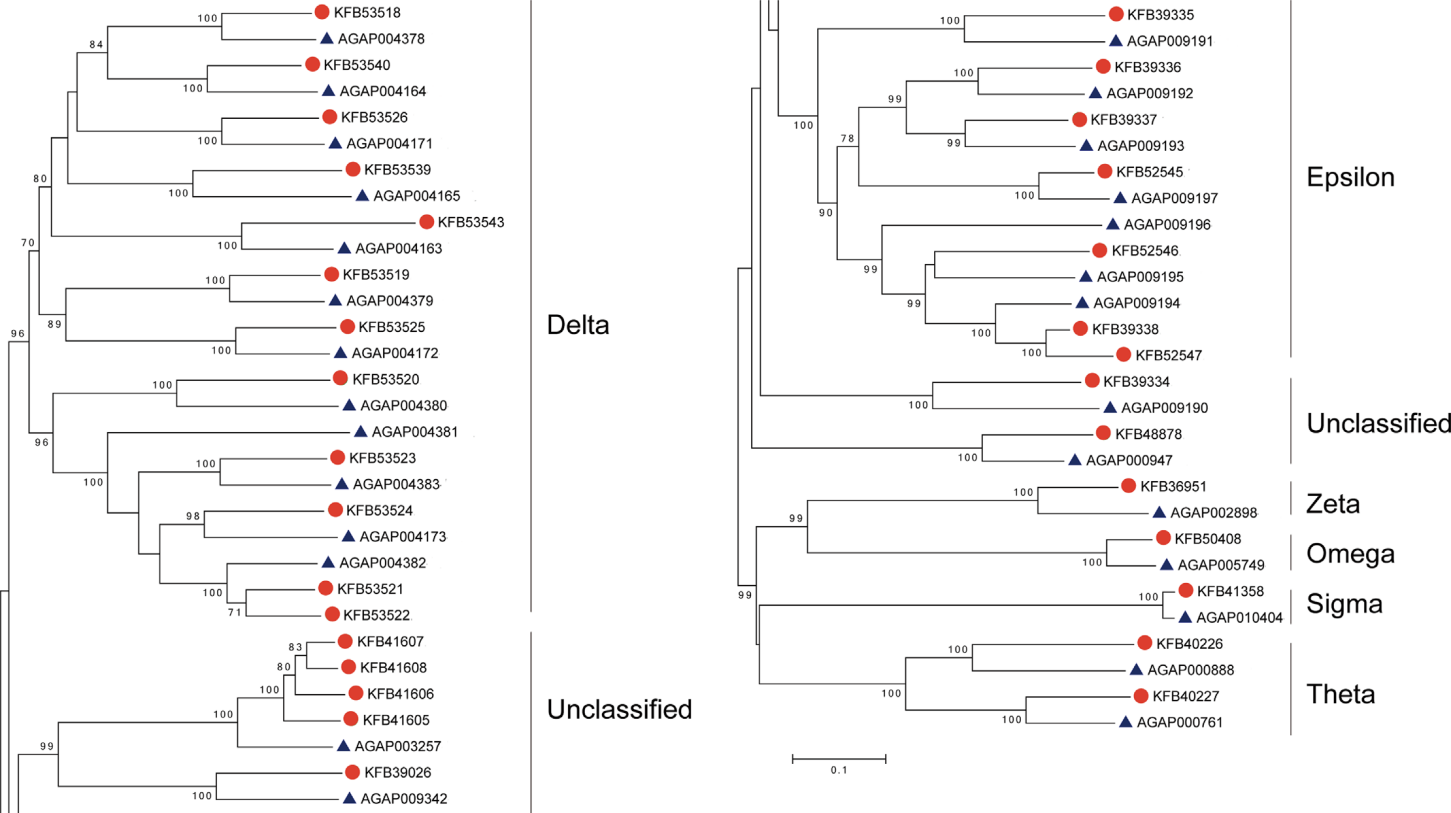
The phylogenetic analysis of glutathione-S-transferases. Unrooted distance neighbor-joining tree showing the phylogeny of glutathione-S-transferases from the genomes of *Anopheles sinensis* (red circle) and *Anopheles gambiae* (blue triangle). The percentage of bootstrap confidence values greater than 70% (1000 replicates) is indicated at the nodes.

A proportion of GSTs, which are as yet unrecognized in the absence of clarifying immunological or biochemical data, were grouped into the Unclassified class. In the present analysis, seven GSTs belonged to the Unclassified class in *An*. *sinensis*. Based on the classification provided by Lumjuan *et al*. [32], some Unclassified GST members could be provisionally classified as two new classes (Xi and Iota classes), which have so far been found uniquely in mosquitoes. The phylogenetic analysis indicated that five Unclassified GSTs (GenBank ID: KFB41605-1608 and KFB39026) as a single clade (99% bootstrap support) may belong to the Xi class. Relative to other mosquito species (one gene per species), multi-copy orthologs with several gene duplications represented an expansion of the Xi class in *An*. *sinensis* (Fig 2). This class of GSTs has been previously implicated in protecting mosquitoes against heme toxicity during blood feeding [54]. A single Unclassified GST (GenBank ID: KFB48878) was identified as belonging to the Iota class, which is the same in in other mosquito species. The remaining Unclassified GST (GenBank ID: KFB39334) observed in *An*. *sinensis*, AsGSTU4, has 1:1:1:1 orthologs in *An*. *gambiae*, *Ae*. *aegypti* and *C*. *quinquefasciatus*. AsGSTU4 was closely associated with the Epsilon class members in the phylogenetic analysis and was located with the majority of the Epsilon class GSTs on the same scaffold (scf7180000695681), which suggested that it should be treated as a member of the Epsilon class. In both *C*. *quinquefasciatus* and *An*. *gambiae*, GSTU4 was classified as an Epsilon GST [55].

The widely distributed, non-insect-specific GSTs showed less duplication in *An*. *sinensis*. Of the remaining genes, two GSTs belonged to the Theta class. Although there are few Theta GSTs, they are highly conserved and were originally thought of as the progenitor class of all GSTs [56]. The ubiquitous Omega, Zeta and Sigma classes were each represented by a single gene in *An*. *sinensis*. The study of *Apis cerana* provided evidence that the expressions of Omega class GSTs could be induced by various abiotic stresses, which suggested that they play protective roles in counteracting oxidative stresses [57]. Zeta class GSTs are widely distributed in nature, from plants to animals [58]. However, this class has not yet been identified in *C*. *quinquefasciatus*. Although a single Sigma GST gene was identified in the genomes of the four species of mosquitoes, alternative splicing in two mosquitoes, *An*. *gambiae* and *Ae*. *aegypti*, increased the number of sigma GST transcripts to two.

#### Resistance associated GSTs

The largest GST classes in *An*. *sinensis* were the insect specific Delta with 12 members. In the present transcriptomic re-analysis, we noticed overexpressed GST mRNAs belonging to the Delta class (Table 2). As noted above, these results were consistent with the concept that that this GST class is frequently involved in insecticide-resistance [59–61]. The best hit homologies of two overexpressed GSTs (GenBank ID: KFB53539 and KFB53540) were to *An*. *gambiae* GSTD2 and GSTD1. GSTD1, which was able to directly detoxify DDT and pyrethroid, play an important role in insecticide metabolism [59, 60, 62]. Here, we suspect the overexpression of GSTD1 participate in pyrethroid resistance in *An*. *sinensis* through the proven GSH conjugation pathway. The expression level of *Drosophila* GSTD2 gene could increase in response to heavy metals, such as cadmium and zinc [63]. It was also interesting to note that GSTD1 and GSTD2 were located next to each other on the same scaffold, suggesting a possible role in resistance for co-expression of these two genes under a common regulatory element.

By contrast, using these same cut-off values, one Theta GST (GenBank ID: KFB40227) was poorly expressed in the *An*. *sinensis* DR-strain (Table 2). The possible role of Theta class in insecticide resistance in insects has been proven. For example, *AcGSTT1-1* is found to bind to organophosphates in *An*. *cracens* [64] and *NlGSTt1* was also insensitive to most insecticides except for chlorpyrifos in *Nilaparvata lugens* [65]. The effect of KFB40227 in insecticide resistance still need to be further studied.

### Carboxyl/cholinesterases

#### Genome level analysis of CCEs

Fifty CCE sequences were identified in *An*. *sinensis* (S3 Table). The classification system described by Oakeshott *et al*. was used to designate the clades in the CCEs phylogeny and this is reproduced in Fig 1C [26]. The insect CCEs fell into three major groups based on their cellular functions: the dietary/detoxification group, the hormone/semiochemical processing group and the neuro/developmental group. These three groups could be further classified into 11 clades: α-esterases (B), integument esterases (D), β-esterases (E), dipteran JH esterases (F), lepidopteran JH esterases (G), glutactins (H), unknown (I), acetylcholinesterases (J), gliotactins (K), neuroligins (L) and neurotactins (M). Ten clades, except D, were identified in *An*. *sinensis*. Clade D includes integumental CCEs implicated in pheromone processing [66]. To date, just one clade D member has been detected in *C*. *quinquefasciatus*, and there is no direct data showing that this clade is represented in other mosquitoes [33].

**Fig 3:**
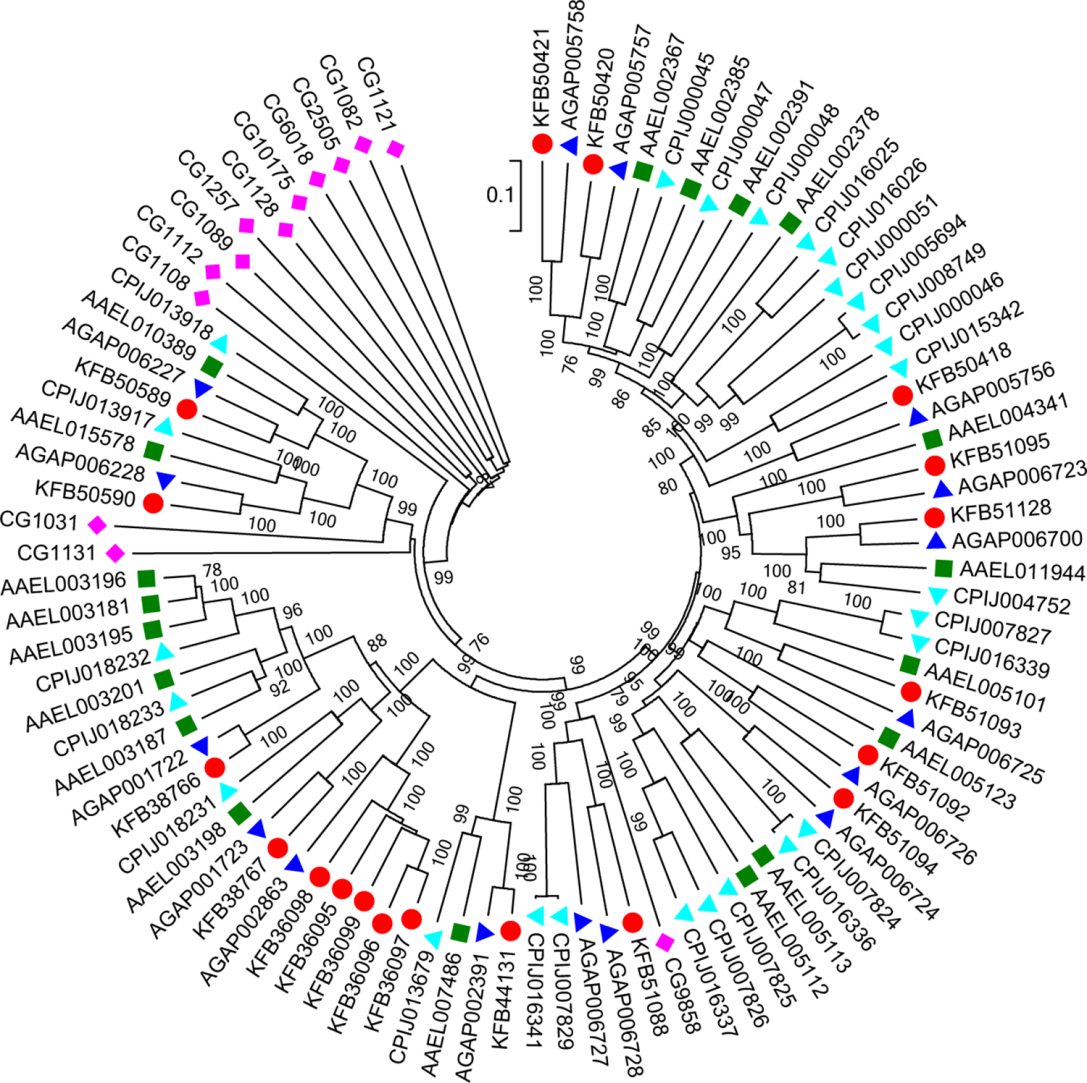
The phylogenetic analysis of α-esterases. Unrooted distance neighbor-joining tree showing the phylogeny of α-esterases from the genomes of *Anopheles sinensis* (red circle), *Anopheles gambiae* (blue triangle), *Aedes aegypti* (green square), *Culex pipiens quinquefasciatusin* (aqua triangle) and *Drosophila melanogaster* (aqua triangle). The percentage of bootstrap confidence values greater than 70% (1000 replicates) is indicated at the nodes.

The dietary/detoxification group contains the A–C clades. Only clade B appeared in Diptera. Compared with other insect orders, α-esterases remained dipteran-specific radiations and had the most members. Among the CCEs identified in the *An*. *sinensis* genome, 44% (22 genes) belong to the α-esterases, which is consistent with other Diptera (30 to 50%). *An*. *sinensis* shows an obvious expansion in one cluster with five members (GenBank ID: KFB36095–36099) (Fig 3). The high level of identity and adjacent genomic locations, together with the lack of clear orthologs in other mosquito species suggested that this might be a rapidly evolving α-esterase cluster. Unlike the rapid radiation of other α-esterases, the esterase A (GenBank ID: KFB50589) and esterase B (GenBank ID: KFB50590) are well conserved and 1:1:1:1 orthologs were found across *An*. *sinensis*, *An*. *gambiae*, *Ae*. *aegypti* and *C*. *quinquefasciatus*. These two esterase genes are encoded by two closely linked genes [67]. In *An*. *sinensis*, the esterase A and esterase B are adjacent genes on the same scaffold, scf7180000696057.

*An*. *sinensis* has 13 members of hormone/semiochemical processing group (D–G clades). The E clade is conserved among the four mosquito species and fruit fly, with two to four β-esterases. The numbers of juvenile hormone esterases (F and G clades) are relatively conserved among the *Aedes* and two *Anopheles* mosquitoes, and are largely different from the expansion found in the *Culex* or the contraction in *Drosophila*. There are fewer neuro/developmental group members (H–M clades) CCEs in *An*. *sinensis* than in the other three mosquito species. This difference is related to a considerable decrease in the level of glutactin, and suggested that genes have been lost during its evolution. Glutactin plays a part in the structure of the envelope of the central nervous system, muscle apodemes and dorsal median cell processes in *D*. *melanogaster* and is thought to be associated with intercellular ordering and adhesion [68]. At present, the role of glutactin in mosquitoes is unclear. For the remaining five clades, the distributions of CCEs were conserved to the extent that almost the same numbers were found in each clade in the four mosquito species, except for clade L in *C*. *quinquefasciatus*. Most insects have two genes encoding acetylcholinesterase (AchE): *ace-1* and *ace-2* [69–79]. These two genes are paralogous and orthologous, respectively, to the *Drosophila ace* gene and arose from ancient gene duplication before the radiation of the Arthropoda.

#### Resistance associated CCEs

Among all expressed CCE transcripts, only two genes were poorly expressed in the *An*. *sinensis* DR-strain, which belonged to E and L clades, respectively. The data available for β-esterases in insects indicate a diversity of functions. The E4 and FE4 esterases were involved in OP resistance in *Myzus persicae* [80]. The *Drosophila* Est6 and Est7 were important in reproductive physiology [81, 82]. The antennal Apo1PDE esterase in silkmoth *Antheraea polyphemus* could degrade sex pheromone [66]. The function of *An*. *sinensis* β-esterases in pyrethroid resistance was unclear and required further investigation. *An*. *sinensis* neuroligins show remarkable conservation among other mosquitoes and *D*. *melanogaster*. Neuroligins are a clade of cell adhesion molecules which participate in bi-directional protein-protein interactions at the synapse [83, 84]. However, the relationship of neuroligins with the pyrethroid resistance phenotype has not been reported.

To our surprise, no overexpressed CCEs was observed in transcription in the *An*. *sinensis* DR-strain. A possible explanation is that CCEs are mainly involved in OP and carbamate resistance. The molecular basis of this resistance mechanism mainly includes the amplification of CCE genes, increased expression or enzymatic activity or mutations in *ace-1* [85–89]. A latest research also showed CCEs played a role in causing a high level of deltamethrin resistance under high insecticide selection pressure in the laboratory stain of *C*. *pipiens pallens* [90]. Whether CCEs are involved in permethrin resistance maybe depends on the level of resistance.

### Insights into diverse detoxification genes across species

The split between subfamilies Anophelinae and Culicinae was estimated as ~122 million years ago, much earlier than the date of the divergence between *An*. *sinensis* and *An*. *gambiae* (~52 million years ago) [27]. Similar numbers of detoxification genes were found in *An*. *sinensis* and *An*. *gambiae*, but just two thirds of those in *Ae*. *aegypti* and two fifths of those in *C*. *quinquefasciatus*. Compared with the Anophelinae, the P450 and CCE supergene families showed pronounced expansion in the Culicinae genome, exhibiting higher rates of sequence divergence. There are several possible explanations for the difference of gene counts in these mosquito species: preference for breeding sites, geographic distribution and vectorial capacity.

*Anopheles* has a preference for clean water habitats, while *Culex* prefers water heavily contaminated with organic material [91]. The expansion of detoxification genes in *C*. *quinquefasciatus* may have played a role in rendering this species particularly adaptable to polluted water.

Both *Ae*. *aegypti* and *C*. *quinquefasciatus* inhabit tropical and subtropical regions throughout the world, whereas *An*. *gambiae* is mainly distributed in sub-Saharan Africa and *An*. *sinensis* is restricted to Southeast Asia. The geographic ranges of *Ae*. *aegypti* and *C*. *quinquefasciatus* are much wider than those of the two *Anopheles* mosquitoes. Varied geographic locations and ecological conditions might have exerted a greater selective pressure on *Ae*. *aegypti* and *C*. *quinquefasciatus* so as to produce a larger repertoire of detoxification genes.

Transmission of arboviruses is largely associated with the *Aedes* and *Culex*, while *Anopheles* is an important vector of human malaria parasites. The molecular mechanisms responsible for the host-parasite interactive relationship differ among the varied species of mosquito vectors and the pathogens they transmit. Besides well-known determinants of vectoral capacity, such as immune and chemosensory genes, detoxification genes may also play roles in this relationship. For example, some P450s were implicated in *C*. *quinquefasciatus*-West Nile virus (CYP6Z12) and *Ae*. *aegypti*-Sindbis and -Dengue virus (CYP6M5) responses, while the expression of juvenile hormone esterase CCEunk7o was modulated during the infection of *Ae*. *aegypti* with *Brugia malayi* [92, 93]. During the long process of evolution, *de novo* origination, gene duplication or loss events have occurred in mosquitoes in response to pathogen infection, which has resulted in the diversification of the related mosquito gene families in compatible host-parasite associations. Exploring the correlation of these detoxification genes with vectoral capacity may provide clues for more detailed investigations of the arthropod vectors of disease.

## Conclusions

It is the first study to analyze the *An*. *sinensis* genome for understanding the molecular mechanisms of insecticide resistance. We identified 174 detoxification genes, comprising 93 P450s, 31 GSTs, and 50 CCEs. The gene number was similar to that in *An*. *gambiae*, but fewer compared with *Aedes aegypti* and *Culex quinquefasciatus*. Transcriptome analysis revealed that at least 124 out of the 174 detoxification genes were expressed in female adult stage. Several P450s and GST genes were oerexpressed in a deltamethrin-resistant strain, indicating that these genes may be involved in pyrethroid resistance.

## Supporting Information

S1 Table Summary of the cytochrome P450 genes in *Anopheles sinensis*. (DOC)

S2 Table Summary of the the glutathione S-transferase genes in *Anopheles sinensis*. (DOC)

S3 Table Summary of the carboxyl/cholin esterases genes in *Anopheles sinensis*. (DOC)
