# Supplementary material for: Genomic Analysis of Detoxification Supergene Families in the Mosquito Anopheles sinensis
Source: PLoS One. 2015 Nov 20;10(11):e0143387. doi: 10.1371/journal.pone.0143387 (PMC4654499; doi:10.1371/journal.pone.0143387)
Supplement: S1 Table — (DOC) [file pone.0143387.s001.doc]

**S1 Table. Summary of the cytochrome P450 genes in *Anopheles sinensis*.**

| Number | Protein (length) | NCBI_ID | Classification | Name | Transcript |
| --- | --- | --- | --- | --- | --- |
| 1 | scf7180000696005.73.protein(503) | KFB45027 | CYP2 | CYP15B1 | Detected |
| 2 | scf7180000695941.10.protein(533) | KFB43110 | CYP2 | CYP18A1 | Detected |
| 3 | scf7180000696058.175.protein(234) | KFB50994 | CYP2 | CYP303A1 | Undetected |
| 4 | scf7180000695910.9.protein(515) | AKH45318 | CYP2 | CYP304B8 | Detected |
| 5 | scf7180000696005.71.protein(504) | KFB45025 | CYP2 | CYP305A16 | Detected |
| 6 | scf7180000696005.72.protein(508) | KFB45026 | CYP2 | CYP305A2 | Detected |
| 7 | scf7180000696050.30.protein(580) | KFB48839 | CYP2 | CYP307A2 | Undetected |
| 8 | scf7180000694985.27.protein(543) | KFB35730 | CYP2 | CYP307B1 | Undetected |
| 9 | scf7180000695236.50.protein(511) | KFB36093 | CYP3 | CYP6AA1 | Detected |
| 10 | scf7180000695236.51.protein(504) | KFB36094 | CYP3 | CYP6AA2 | Detected |
| 11 | scf7180000695236.61.protein(500) | KFB36104 | CYP3 | CYP6AD1 | Undetected |
| 12 | scf7180000696029.23.protein(493) | KFB46763 | CYP3 | CYP6AF3 | Detected |
| 13 | scf7180000694399.18.protein(501) | KFB35513 | CYP3 | CYP6AG1 | Detected |
| 14 | scf7180000694399.17.protein(504) | KFB35512 | CYP3 | CYP6AG2 | Detected |
| 15 | scf7180000696060.357.protein(499) | KFB51698 | CYP3 | CYP6AH4 | Detected |
| 16 | scf7180000696060.358.protein(499) | KFB51699 | CYP3 | CYP6AH5 | Detected |
| 17 | scf7180000696112.5.protein(514) | KFB52629 | CYP3 | CYP6AJ1 | Undetected |
| 18 | scf7180000696112.12.protein(183) | KFB52635 | CYP3 | CYP6AK1 N-term fragment | Undetected |
| 19 | scf7180000696112.11.protein(114) | KFB52634 | CYP3 | CYP6AK2P N-term fragment | Undetected |
| 20 | scf7180000695502.2.protein(355) | KFB36867 | CYP3 | CYP6M1 missing C-term | Detected |
|  | scf7180000695502.3.protein(151) | KFB36868 |  | CYP6M1 C-term fragment |  |
| 21 | scf7180000695502.5.protein(499) | KFB36870 | CYP3 | CYP6M17 | Detected |
| 22 | scf7180000695502.6.protein(495) | KFB36871 | CYP3 | CYP6M18 | Detected |
| 23 | scf7180000695502.7.protein(501) | KFB36872 | CYP3 | CYP6M2 | Detected |
| 24 | scf7180000695935.4.protein(252) | KFB42894 | CYP3 | CYP6M3 C-term fragment | Detected |
|  | scf7180000695935.5.protein(111) | KFB42895 |  | CYP6M3 N-term fragment |  |
| 25 | scf7180000695502.4.protein(500) | KFB36869 | CYP3 | CYP6N1 | Detected |
| 26 | scf7180000695685.4.protein(501) | KFB39403 | CYP3 | CYP6N2 | Detected |
| 27 | scf7180000695502.1.protein(495) | KFB36866 | CYP3 | CYP6N28 | Detected |
| 28 | scf7180000695236.59.protein(525) | KFB36102 | CYP3 | CYP6P1 | Detected |
| 29 | scf7180000695236.60.protein(510) | KFB36103 | CYP3 | CYP6P2 | Detected |
| 30 | scf7180000695236.58.protein(509) | KFB36101 | CYP3 | CYP6P4 | Detected |
| 31 | scf7180000695685.5.protein(489) | KFB39404 | CYP3 | CYP6HP1 | Detected |
| 32 | scf7180000695685.6.protein(500) | KFB39405 | CYP3 | CYP6S1 | Detected |
| 33 | scf7180000695685.1.protein(116) | - | CYP3 | CYP6Y1 N-term fragment | Detected |
|  | scf7180000695685.2.protein(502) | KFB39401 |  | CYP6Y1 missing N-term |  |
| 34 | scf7180000695685.3.protein(526) | KFB39402 | CYP3 | CYP6Y2 | Detected |
| 35 | scf7180000696007.3.protein(460) | KFB45126 | CYP3 | CYP6Z22 | Detected |
| 36 | scf7180000696007.2.protein(389) | KFB45125 | CYP3 | CYP6Z21 | Detected |
| 37 | scf7180000695502.12.protein(242) | KFB36876 | CYP3 | CYP6Z19 C-term fragment | Detected |
|  | scf7180000695502.13.protein(182) | KFB36877 |  | CYP6Z19 N-term fragment |  |
| 38 | scf7180000695721.1.protein(496) | AKH45321 | CYP3 | CYP6Z20 | Detected |
| 39 | scf7180000695502.11.protein(491) | KFB36875 | CYP3 | CYP6Z18 | Detected |
| 40 | scf7180000696055.159.protein(547) | KFB49807 | CYP3 | CYP9J56 | Detected |
| 41 | scf7180000696055.158.protein(541) | KFB49806 | CYP3 | CYP9J55 | Detected |
| 42 | scf7180000696055.148.protein(540) | KFB49796 | CYP3 | CYP9J49 | Detected |
| 43 | scf7180000696055.149.protein(538) | KFB49797 | CYP3 | CYP9J50 | Detected |
| 44 | scf7180000696055.150.protein(504) | KFB49798 | CYP3 | CYP9J51 | Detected |
| 45 | scf7180000696055.151.protein(541) | KFB49799 | CYP3 | CYP9J52 | Detected |
| 46 | scf7180000696055.152.protein(541) | KFB49800 | CYP3 | CYP9J53 | Detected |
| 47 | scf7180000696055.155.protein(541) | KFB49803 | CYP3 | CYP9J54 | Detected |
| 48 | scf7180000696079.4.protein(511) | KFB52084 | CYP3 | CYP9K1 | Detected |
| 49 | scf7180000696055.156.protein(534) | KFB49804 | CYP3 | CYP9L5 | Detected |
| 50 | scf7180000696055.157.protein(537) | KFB49805 | CYP3 | CYP9L6 | Detected |
| 51 | scf7180000695681.39.protein(530) | KFB38993 | CYP3 | CYP9M1 | Detected |
| 52 | scf7180000696232.2.protein(407) | AKH45322 | CYP3 | CYP9M2 | Detected |
| 53 | scf7180000695972.21.protein(499) | KFB43911 | CYP4 | CYP325A4 | Undetected |
| 54 | scf7180000695972.22.protein(437) | KFB43912 | CYP4 | CYP325B2 | Undetected |
| 55 | scf7180000695939.68.protein(195) | KFB42968 | CYP4 | CYP325C4 C-term fragment | Detected |
|  | scf7180000695939.69.protein(205) | KFB42969 |  | CYP325C4 N-term fragment |  |
| 56 | scf7180000695972.23.protein(363) | KFB43913 | CYP4 | CYP325C5 | Undetected |
| 57 | scf7180000696076.13.protein(503) | KFB52027 | CYP4 | CYP325F2 | Detected |
| 58 | scf7180000696076.10.protein(413) | KFB52024 | CYP4 | CYP325F3 | Detected |
| 59 | scf7180000696076.11.protein(362) | KFB52025 | CYP4 | CYP325F4 | Detected |
| 60 | scf7180000696076.12.protein(126) | KFB52026 | CYP4 | CYP325F5P N-term fragment | Undetected |
| 61 | scf7180000696076.14.protein(509) | KFB52028 | CYP4 | CYP325G1 | Detected |
| 62 | scf7180000696124.32.protein(501) | KFB53108 | CYP4 | CYP325H1 | Undetected |
| 63 | scf7180000695800.6.protein(482) | KFB41382 | CYP4 | CYP325J1 | Undetected |
| 64 | scf7180000696127.10.protein(513) | KFB53238 | CYP4 | CYP325K1 | Undetected |
| 65 | scf7180000687771.2.protein(293) | AKH45320 | CYP4 | CYP4AA1 C-term fragment | Detected |
|  | scf7180000694122.4.protein(181) | - |  | CYP4AA1 N-term fragment |  |
| 66 | scf7180000695750.28.protein(492) | KFB40893 | CYP4 | CYP4AR1 | Undetected |
| 67 | scf7180000696079.197.protein(544) | KFB52268 | CYP4 | CYP4C25 | Detected |
| 68 | scf7180000696079.193.protein(538) | KFB52264 | CYP4 | CYP4C26 | Detected |
| 69 | scf7180000696054.77.protein(506) | KFB49529 | CYP4 | CYP4C27 | Detected |
| 70 | scf7180000696079.194.protein(532) | KFB52265 | CYP4 | CYP4C28 | Detected |
| 71 | scf7180000696054.83.protein(523) | KFB49535 | CYP4 | CYP4C35 | Detected |
| 72 | scf7180000696079.192.protein(537) | KFB52263 | CYP4 | CYP4C37 | Detected |
| 73 | scf7180000695750.29.protein(513) | KFB40894 | CYP4 | CYP4D15 | Detected |
| 74 | scf7180000692944.1.protein(187) | - | CYP4 | CYP4D16 N-term fragment | Undetected |
| 75 | scf7180000695750.32.protein(503) | KFB40897 | CYP4 | CYP4D22 | Undetected |
| 76 | scf7180000695709.11.protein(565) | KFB40088 | CYP4 | CYP4G16 | Detected |
| 77 | scf7180000695865.289.protein(556) | KFB42070 | CYP4 | CYP4G17 | Detected |
| 78 | scf7180000695742.43.protein(505) | KFB40666 | CYP4 | CYP4H14 | Detected |
| 79 | scf7180000695742.53.protein(130) | KFB40676 | CYP4 | CYP4H25 C-term fragment | Detected |
|  | scf7180000695742.51.protein(211) | KFB40674 |  | CYP4H25 N-term fragment |  |
| 80 | scf7180000696013.46.protein(506) | KFB45597 | CYP4 | CYP4H17 | Detected |
| 81 | scf7180000695742.54.protein(503) | KFB40677 | CYP4 | CYP4H45 | Detected |
| 82 | scf7180000696005.34.protein(515) | KFB44988 | CYP4 | CYP4J10 | Detected |
| 83 | scf7180000696005.33.protein(531) | KFB44987 | CYP4 | CYP4J5 | Detected |
| 84 | scf7180000696005.31.protein(382) | KFB44985 | CYP4 | CYP4J9a | Detected |
| 85 | scf7180000696005.32.protein(186) | KFB44986 | CYP4 | CYP4J9b | Undetected |
| 86 | scf7180000695750.26.protein(265) | KFB40891 | CYP4 | CYP4K4 N-term fragment | Detected |
|  | scf7180000695750.27.protein(222) | KFB40892 |  | CYP4K4 C-term fragment |  |
| 87 | scf7180000694298.7.protein(519) | KFB35468 | mitochondrial CYP | CYP12F15 | Detected |
| 88 | scf7180000694298.8.protein(529) | KFB35469 | mitochondrial CYP | CYP12F16 | Detected |
| 89 | scf7180000694298.9.protein(524) | AKH45317 | mitochondrial CYP | CYP12F17 | Detected |
| 90 | scf7180000694298.6.protein(534) | KFB35467 | mitochondrial CYP | CYP12F18 | Detected |
| 91 | scf7180000695971.76.protein(496) | KFB43847 | mitochondrial CYP | CYP302A1 | Undetected |
| 92 | scf7180000688285.1.protein(159) | AKH45319 | mitochondrial CYP | CYP314A1 C-term fragment | Undetected |
| 93 | scf7180000695702.40.protein(440) | KFB39666 | mitochondrial CYP | CYP315A1 | Detected |
