# Supplementary material for: Genomic Analysis of Detoxification Supergene Families in the Mosquito Anopheles sinensis
Source: PLoS One. 2015 Nov 20;10(11):e0143387. doi: 10.1371/journal.pone.0143387 (PMC4654499; doi:10.1371/journal.pone.0143387)
Supplement: S2 Table — (DOC) [file pone.0143387.s002.doc]

**S2 Table. Summary of the the glutathione S-transferase genes in *Anopheles sinensis*.**

| Number | Protein (length) | NCBI_ID | Classification | Transcript |
| --- | --- | --- | --- | --- |
| 1 | scf7180000696131.131.protein(211) | KFB53523 | Delta | Undetected |
| 2 | scf7180000696131.126.protein(213) | KFB53518 | Delta | Detected |
| 3 | scf7180000696131.128.protein(210) | KFB53520 | Delta | Detected |
| 4 | scf7180000696131.151.protein(247) | KFB53543 | Delta | Undetected |
| 5 | scf7180000696131.129.protein(210) | KFB53521 | Delta | Detected |
| 6 | scf7180000696131.133.protein(218) | KFB53525 | Delta | Undetected |
| 7 | scf7180000696131.127.protein(280) | KFB53519 | Delta | Detected |
| 8 | scf7180000696131.134.protein(215) | KFB53526 | Delta | Undetected |
| 9 | scf7180000696131.148.protein(201) | KFB53540 | Delta | Detected |
| 10 | scf7180000696131.130.protein(195) | KFB53522 | Delta | Detected |
| 11 | scf7180000696131.132.protein(214) | KFB53524 | Delta | Undetected |
| 12 | scf7180000696131.147.protein(209) | KFB53539 | Delta | Detected |
| 13 | scf7180000696106.100.protein(222) | KFB52546 | Epsilon | Detected |
| 14 | scf7180000696106.98.protein(223) | KFB52545 | Epsilon | Detected |
| 15 | scf7180000695681.391.protein(223) | KFB39335 | Epsilon | Detected |
| 16 | scf7180000696106.101.protein(206) | KFB52547 | Epsilon | Detected |
| 17 | scf7180000695681.393.protein(223) | KFB39337 | Epsilon | Detected |
| 18 | scf7180000695681.392.protein(224) | KFB39336 | Epsilon | Detected |
| 19 | scf7180000695681.394.protein(221) | KFB39338 | Epsilon | Detected |
| 20 | scf7180000696056.125.protein(247) | KFB50408 | Omega | Detected |
| 21 | scf7180000695799.13.protein(203) | KFB41358 | Sigma | Detected |
| 22 | scf7180000695709.151.protein(237) | KFB40226 | Theta | Detected |
| 23 | scf7180000695709.152.protein(208) | KFB40227 | Theta | Detected |
| 24 | scf7180000695821.5.protein(222) | KFB41607 | Unclassified | Detected |
| 25 | scf7180000695821.6.protein(322) | KFB41608 | Unclassified | Detected |
| 26 | scf7180000695821.3.protein(230) | KFB41605 | Unclassified | Detected |
| 27 | scf7180000695681.73.protein(218) | KFB39026 | Unclassified | Detected |
| 28 | scf7180000695821.4.protein(222) | KFB41606 | Unclassified | Detected |
| 29 | scf7180000696050.71.protein(208) | KFB48878 | Unclassified | Detected |
| 30 | scf7180000695681.390.protein(222) | KFB39334 | Unclassified | Detected |
| 31 | scf7180000695504.76.protein(172) | KFB36951 | Zeta | Undetected |
