# Supplementary material for: Genomic Analysis of Detoxification Supergene Families in the Mosquito Anopheles sinensis
Source: PLoS One. 2015 Nov 20;10(11):e0143387. doi: 10.1371/journal.pone.0143387 (PMC4654499; doi:10.1371/journal.pone.0143387)
Supplement: S3 Table — (DOC) [file pone.0143387.s003.doc]

**S3 Table. Summary of the carboxyl/cholin esterases genes in *Anopheles sinensis*.**

| Number | Protein (length) | NCBI_ID | Classification | Transcript |
| --- | --- | --- | --- | --- |
| 1 | scf7180000696057.55.protein(541) | KFB50589 | B | Detected |
| 2 | scf7180000696057.56.protein(535) | KFB50590 | B | Detected |
| 3 | scf7180000695678.51.protein(566) | KFB38767 | B | Undetected |
| 4 | scf7180000695977.32.protein(574) | KFB44131 | B | Undetected |
| 5 | scf7180000696058.275.protein(527) | KFB51093 | B | Undetected |
| 6 | scf7180000695236.55.protein(557) | KFB36098 | B | Detected |
| 7 | scf7180000696058.271.protein(104) | KFB51089 | B | Undetected |
| 8 | scf7180000696058.273.protein(99) | KFB51091 | B | Detected |
| 9 | scf7180000695236.54.protein(469) | KFB36097 | B | Detected |
| 10 | scf7180000696058.277.protein(582) | KFB51095 | B | Undetected |
| 11 | scf7180000695236.52.protein(554) | KFB36095 | B | Detected |
| 12 | scf7180000696058.276.protein(606) | KFB51094 | B | Detected |
| 13 | scf7180000696056.135.protein(580) | KFB50418 | B | Undetected |
| 14 | scf7180000696058.274.protein(549) | KFB51092 | B | Detected |
| 15 | scf7180000696058.272.protein(445) | KFB51090 | B | Detected |
| 16 | scf7180000695236.53.protein(1725) | KFB36096 | B | Detected |
| 17 | scf7180000696058.270.protein(418) | KFB51088 | B | Undetected |
| 18 | scf7180000696056.138.protein(587) | KFB50421 | B | Detected |
| 19 | scf7180000696058.310.protein(595) | KFB51128 | B | Undetected |
| 20 | scf7180000695236.56.protein(556) | KFB36099 | B | Detected |
| 21 | scf7180000695678.50.protein(562) | KFB38766 | B | Undetected |
| 22 | scf7180000696056.137.protein(587) | KFB50420 | B | Detected |
| 23 | scf7180000696049.274.protein(1656) | KFB48754 | E | Detected |
| 24 | scf7180000696049.273.protein(567) | KFB48753 | E | Detected |
| 25 | scf7180000696049.38.protein(586) | KFB48521 | F | Undetected |
| 26 | scf7180000696049.39.protein(594) | KFB48522 | F | Detected |
| 27 | scf7180000696049.41.protein(580) | KFB48524 | F | Detected |
| 28 | scf7180000695970.90.protein(203) | KFB43647 | F | Undetected |
| 29 | scf7180000696049.40.protein(570) | KFB48523 | F | Detected |
| 30 | scf7180000696018.9.protein(512) | AKH45323 | F | Undetected |
| 31 | scf7180000686859.1.protein(144) | - | F | Undetected |
| 32 | scf7180000695747.15.protein(522) | KFB40848 | G | Detected |
| 33 | scf7180000695747.2.protein(510) | KFB40837 | G | Undetected |
| 34 | scf7180000695747.16.protein(612) | KFB40849 | G | Detected |
| 35 | scf7180000695944.9.protein(625) | AKH45324 | G | Undetected |
| 36 | scf7180000696037.32.protein(553) | KFB47223 | H | Undetected |
| 37 | scf7180000696038.52.protein(1293) | KFB47281 | H | Detected |
| 38 | scf7180000696038.51.protein(552) | KFB47280 | H | Detected |
| 39 | scf7180000696037.31.protein(601) | KFB47222 | H | Undetected |
| 40 | scf7180000696049.312.protein(641) | KFB48792 | I | Undetected |
| 41 | scf7180000695680.150.protein(646) | KFB38925 | J | Detected |
| 42 | scf7180000695643.3.protein(361) | KFB38318 | J C-term fragment | Detected |
|  | scf7180000692126.2.protein(385) | KFB35326 | J N-term fragment |  |
| 43 | scf7180000695536.9.protein(351) | - | K fragment | Undetected |
| 44 | scf7180000692630.1.protein(178) | KFB35340 | L | Undetected |
| 45 | scf7180000694929.1.protein(75) | KFB35650 | L | Undetected |
| 46 | scf7180000696055.402.protein(931) | KFB50048 | L | Undetected |
| 47 | scf7180000696126.40.protein(859) | KFB53170 | L | Undetected |
| 48 | scf7180000692630.3.protein(1375) | KFB35342 | L | Detected |
| 49 | scf7180000695570.17.protein(638) | KFB38131 | M | Undetected |
| 50 | scf7180000696057.75.protein(900) | KFB50608 | M | Detected |
